# Power in pairs: assessing the statistical value of paired samples in tests for differential expression

**DOI:** 10.1186/s12864-018-5236-2

**Published:** 2018-12-20

**Authors:** John R. Stevens, Jennifer S. Herrick, Roger K. Wolff, Martha L. Slattery

**Affiliations:** 1 Department of Mathematics and Statistics, Utah State University, Logan, UT USA; 2 Division of Epidemiology, Department of Internal Medicine, University of Utah, Salt Lake City, UT USA

**Keywords:** Study design, Statistical power, RNA-Seq, Microarray, microRNA

## Abstract

**Background:**

When genomics researchers design a high-throughput study to test for differential expression, some biological systems and research questions provide opportunities to use paired samples from subjects, and researchers can plan for a certain proportion of subjects to have paired samples. We consider the effect of this paired samples proportion on the statistical power of the study, using characteristics of both count (RNA-Seq) and continuous (microarray) expression data from a colorectal cancer study.

**Results:**

We demonstrate that a higher proportion of subjects with paired samples yields higher statistical power, for various total numbers of samples, and for various strengths of subject-level confounding factors. In the design scenarios considered, the statistical power in a fully-paired design is substantially (and in many cases several times) greater than in an unpaired design.

**Conclusions:**

For the many biological systems and research questions where paired samples are feasible and relevant, substantial statistical power gains can be achieved at the study design stage when genomics researchers plan on using paired samples from the largest possible proportion of subjects. Any cost savings in a study design with unpaired samples are likely accompanied by underpowered and possibly biased results.

**Electronic supplementary material:**

The online version of this article (10.1186/s12864-018-5236-2) contains supplementary material, which is available to authorized users.

## Background

When a genomics research team is planning a study that will involve testing multiple biomarkers (or features) for differential expression, many important decisions must be made at this study design stage. Previous literature has addressed replication and statistical analysis plans (see, for example, [1–6]). Often the test for differential expression is between two conditions (say tumor and normal tissue) that can be, but are not always, sampled within each subject. When a given subject provides samples from both conditions, it can be said that this subject has paired samples. In cancer genomics research studies, the proportion of subjects with paired samples can be as high as 1 [7, 8] or reasonably close to 1 [9], but is most often zero or essentially zero [10–12], as has been discussed previously [13].

At an extreme, normal tissue sample expression from a database [14] could be compared to expression in study-derived tumor tissue samples. Such an approach would certainly introduce the risk of substantial batch effects, the statistical consequences of which have been addressed previously [15]. Specifically, the tumor vs. normal comparison of interest would be completely confounded with the study vs. database batch effect. Even if some normalization approach were able to successfully remove or account for these batch effects, the corresponding data would still be comprised of entirely unpaired samples.

Over the past several years, the genomics literature has included attention to principles of good experimental design for a variety of genomics platforms [5, 16, 17]. However, the design-stage choice of the proportion of subjects with paired samples has not received attention in the genomics literature, despite its potential impact on overall statistical power of the study.

Statistical power in this context refers to the probability that truly differentially expressed features will be called statistically significant. Higher statistical power can be achieved through a combination of good study design and appropriate statistical analysis plan [5, 16–18]. One element of study design is the choice of a proportion of subjects with paired samples.

The purpose of this manuscript is to draw attention to (and to quantify) the statistical value of paired samples in genomics studies where such paired samples are feasible. We use a hypothetical (but realistically recurring) scenario of a genomics research team that, with the reality of limited resources, has determined that they can afford to run N samples for a given study. In designing this study, the team can collect paired samples on some percentage of subjects in the study; for the sake of conciseness, we assume the remaining subjects with unpaired samples will be evenly split between conditions (tumor and normal, for example), for a total of N samples. (Such balance is known to generally provide higher statistical power.)

We evaluate the statistical power in tests for differential expression, under a variety of scenarios (number of samples N and percent of subjects with paired samples), for both count (such as RNA-Seq) and continuous (such as microarray-based) expression data. The power evaluations are made using the characteristics of microarray-based miRNA and RNA-Seq-based gene expression data from a large colorectal cancer (CRC) study (over 2000 miRNAs on each of approximately 2000 subjects, and over 17,000 genes [using RNA-Seq] on each of over 200 subjects) that included a majority of subjects having paired samples (both tumor and normal). This colorectal cancer study and the power evaluation approaches are described in the Methods section below.

We highlight the role of possibly confounding subject-level factors, which are subject characteristics that affect expression values in both normal and tumor samples. Examples of such possible confounding factors include dietary or lifestyle factors. Additionally, tissue samples may vary between hospitals in how they are handled during processing as well as pathological observations, introducing additional sources of potential bias in non-paired data. We demonstrate that in the presence of such confounding factors, a higher proportion of subjects with paired samples leads to a considerable gain in statistical power. In addition, using our CRC data we demonstrate the substantial power gain that we observe when comparing an analysis of paired samples to an analysis of unpaired samples.

## Results

**Fig. 1:**
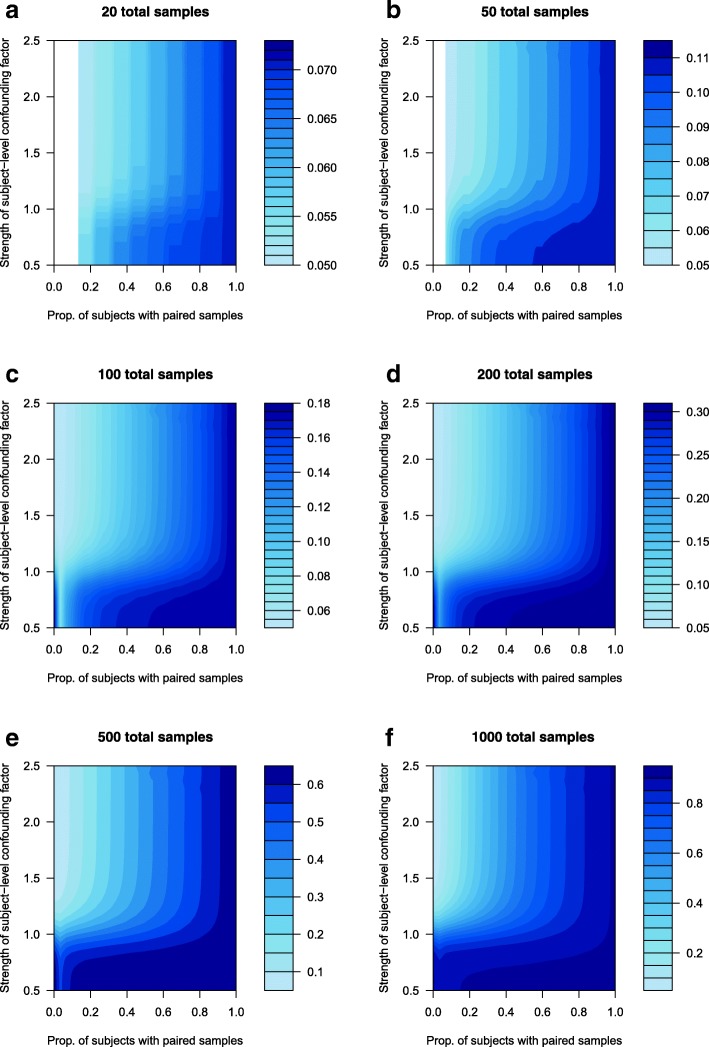
Approximate power contours for count data, such as RNA-Seq. The power range varies with sample size, as indicated in the legend to the right of each sub-figure

**Fig. 2:**
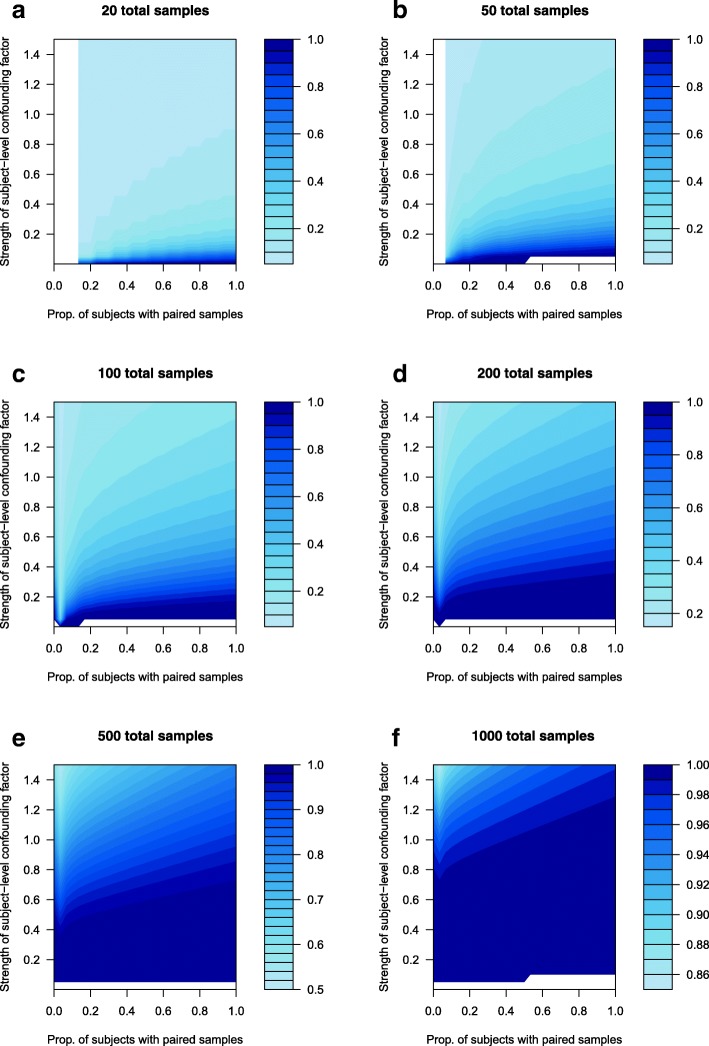
Approximate power contours for continuous data, such as microarray-based. The power range varies with sample size, as indicated in the legend to the right of each sub-figure

Figure 1 visualizes approximate power contours for count expression data (as for RNA-Seq data), and Fig. 2 visualizes approximate power contours for continuous expression data (as for microarray data). In both data types, the strength of the confounding factor is related to the variance of the subject effect in an appropriate generalized linear mixed model (as described in the Methods section below). The power evaluation in Figs. 1 and 2 is based on detecting a fold change of 1.25 in RNA-Seq data and 1.5 in microarray data, respectively. These fold change values as well as the figures’ ranges of the strength of the confounding factor are based on observed estimates from the colorectal cancer study data, as described in the Methods section. (When alternative fold change values were used, the same basic trends were seen as in Figs. 1 and 2; only results for fold change values of 1.25 in RNA-Seq data (Fig. 1) and 1.5 in microarray data (Fig. 2) are reported, in order to focus the presentation and discussion of results.) The blank regions at the far left in Figs. 1a-b and 2a-b result from numerical problems for lower sample sizes when very low proportions of subjects have paired samples; for these cases no power approximations could be made. Similar numerical problems result in no power approximations in Fig. 2 for some scenarios with very low strengths of subject-level confounding factors.

While the overall power in Figs. 1 and 2 increases for larger numbers of samples (as would be expected), the central message of these results is that power also generally increases for a higher proportion of subjects with paired samples, for any given number of total samples. One unexpected result in Figs. 1 and 2 is that as the proportion of subjects with paired samples increases from zero, there can be a slight dip in power when only around 5 % of subjects have paired samples. This is addressed in the Discussion section below.

Figures 1 and 2 also show that statistical power (to detect the treatment effect) decreases as the strength of the subject-level confounding factor increases; this is due to a larger subject-level confounding factor effectively obscuring the true treatment effect. This loss of power is especially noticeable for lower sample sizes and for lower proportions of subjects with paired samples.

Figures 1 and 2 illustrate that for any given strength of confounding factor, and for a given total number of samples, the highest statistical power is obtained when all subjects have paired samples. Having fewer than all subjects with paired samples results in a loss of power, even for larger sample sizes, and particularly for greater strength of subject-level confounding factors. Even for the largest sample sizes considered here, there is a clear power gain with a higher proportion of subjects with paired samples. While the gain may appear less for some lower strengths of subject-level confounding factors (as in Fig. 1e and f), it is important to keep in mind that each gene can have its own subject-level confounding factors (as subject-level lifestyle and genetic factors can affect expression of specific genes), and it is not known a priori what the strength of their effect is. As such, it cannot be known which genes will experience the greatest (or least) statistical power boost by having a higher proportion of subjects with paired samples, but it is clear that overall, there is a (often substantial) statistical power gain to be had in study designs with higher proportions of subjects with paired samples.

The power approximations in Figs. 1 and 2 are based on the probability distribution method using properties of previous RNA-Seq and microarray studies (see Methods section and code in Additional files 1 and 2). Similar power contours as well as general control of the false discovery rate (FDR) [19] were obtained from the simulation method (see Methods section) in Additional files 3 and 4, for continuous expression data.

**Fig. 3:**
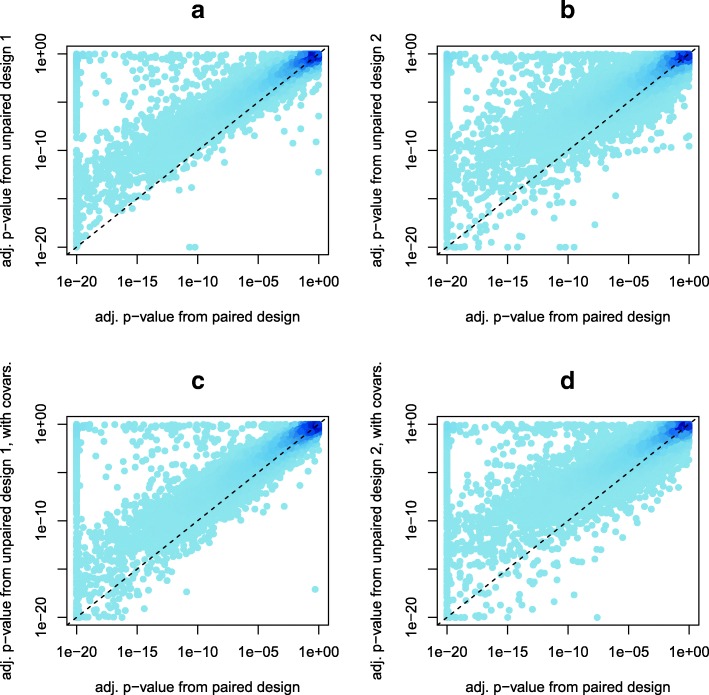
Comparison of FDR-adjusted *p*-values in RNA-Seq data from colon cancer study, for paired design and two unpaired designs, considering without covariates (a-b) and with covariates (c-d) in the analysis of the unpaired designs. All axes’ tick marks are spaced on the log scale

Figure 3 gives a glimpse at the power gain by paired analysis in our CRC study. Local point density is represented by color in Fig. 3, with darker colors corresponding to greater density; this was achieved using the grDevices package in R [20]. Compared to the analysis of a fully-paired design (horizontal axis in all Fig. 3 panels), the two unpaired designs considered here exhibited a clear loss of statistical power (evidenced by the abundance of points above the reference line of equality in Fig. 3a-d). In general, the paired design resulted in more significant tests (smaller FDR-adjusted *p*-values). In particular, whereas the paired design resulted in 8856 (of 17,462) features called significantly differentially expressed at FDR .01, unpaired designs 1 and 2 resulted in 6865 and 7076 significant features, respectively. Accounting for covariates did mitigate the loss of power somewhat in unpaired design 1, resulting in 7192 significant features; but accounting for covariates in unpaired design 2 actually resulted in only 6755 significant features.

**Fig. 4:**
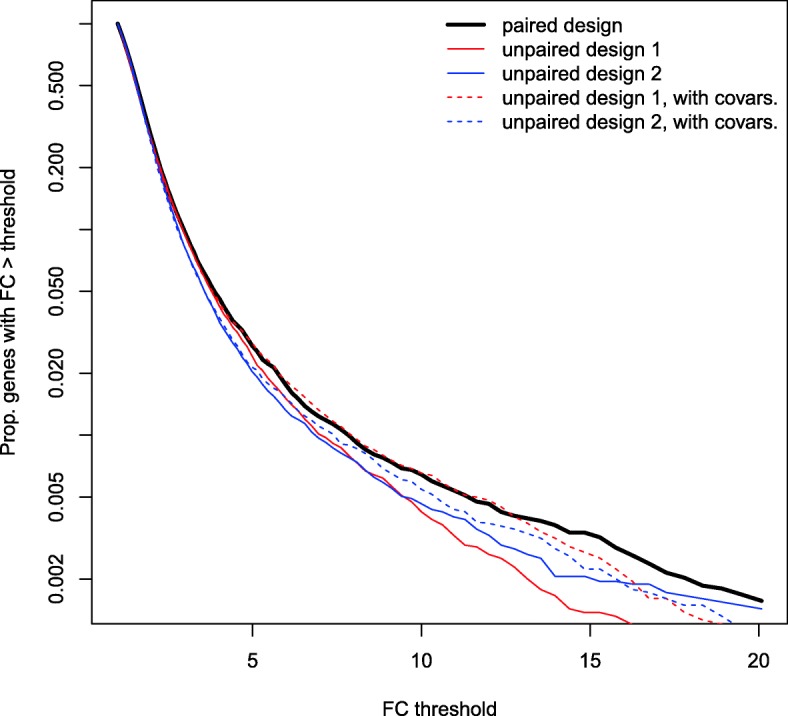
Proportion of genes exceeding given fold change (FC) thresholds in RNA-Seq data from colon cancer study, for paired design and two unpaired designs, considering without covariates (solid red/blue) and with covariates (dashed red/blue) in the analysis of the unpaired designs. Vertical axis’s tick marks are spaced on the log scale

Figure 4 illustrates another advantage of the paired design over the unpaired designs in our CRC study, beyond statistical power. When a simple fold change threshold is considered, the paired design tends to result in greater fold changes, in the sense that a higher proportion of genes will have fold changes above a given threshold in the paired design than in the unpaired designs considered. This systematic trend in Fig. 4 becomes clearer for larger fold change thresholds. “Fold change” here is in absolute magnitude, so that both up- and down-regulation are represented. This modest underestimation of magnitude of fold change in the unpaired designs is mitigated somewhat by accounting for covariates.

## Discussion

The only exception to the “higher proportion of subjects with paired samples yields higher statistical power” result seen in Figs. 1 and 2 occurred for around 5 % of subjects with paired samples in some figure panels, when there was a low strength of subject-level confounding factor. This is likely due to a degree of freedom issue. When there are no subjects with paired samples, then there are no random subject effects to estimate in the model. For every subject with paired samples, a random subject effect must be estimated, which effectively costs one degree of freedom. In general, losing these degrees of freedom reduces overall statistical power somewhat. However, when a large enough proportion of subjects have paired samples, the confounding factors are effectively estimated out, leaving a clearer picture of the true underlying treatment effect, and so statistical power is increased. When only a small handful of subjects (such as around 5 %) have paired samples, then the degrees of freedom must be spent to estimate their subject effects in the model, but it seems that, at such a low proportion of subjects with paired samples, there is not yet sufficient information on the confounding factors’ effects to effectively estimate them out, leaving the treatment effect still somewhat obscured. This degree of freedom expense (and its negative effect on power) appears to be mitigated when at least 10 % of subjects have paired samples, or when the strength of the subject-level confounding factor is higher.

If fewer than about 10 % of subjects have paired samples and there is very weak subject-level confounding, then from a statistical power perspective it may be tempting to drop one of each subject’s paired samples, and run an analysis on fully unpaired data. However, this will lower the effective sample size, further reducing statistical power. Without clear a priori knowledge of the magnitude of subject-level confounding in a planned study, the best strategy would be to plan for as high a proportion as possible of subjects with paired samples, and use all valid samples (even if unpaired) that are available.

The comparison of paired and unpaired designs in our CRC data yielded clear evidence that having all subjects with paired samples results in a gain in statistical power compared to having all unpaired samples. For example, the adjusted *p*-values in the paired design analysis tended to be systematically smaller than in the unpaired designs’ analyses (Fig. 3), resulting in more significantly differentially expressed features (over 8800 in the paired design compared to about 6800–7200 in the unpaired designs considered). Somewhat surprising was the discrepancy in power loss mitigation when accounting for subject-level covariates in an unpaired design. While accounting for a few covariates did result in a modest gain in power for unpaired design 1 (going from 6865 significant features without covariates to 7192 with covariates), such accounting actually reduced the number of significant features in unpaired design 2 (from 7076 to 6755). This suggests that depending on the situation (and available subject-level covariates), even when several subject-level covariates are available, accounting for them may or may not increase statistical power, but in any case it does not seem likely that using subject-level covariates could achieve the same statistical power gains that are possible with the simple strategy of pairing samples for a high proportion of subjects. In addition, a major limitation of accounting for subject-level covariates with unpaired data is that it is usually not known which diet, lifestyle, or genetic factor(s) may be confounding for any given gene.

Another potential risk in an unpaired design is the underestimation of the magnitude of differential expression (Fig. 4). In our CRC data there were a number of genes with fold changes greater than 1.5 in an analysis of paired data, but that had fold changes less than 1.3 when analyzed as unpaired data. While the majority of our focus is on the statistical power gains to be had for higher proportions of subjects with paired data, we also point out that from the perspective of using a “meaningful difference” threshold in addition to statistical significance, even moderate underestimation of magnitude of fold change (as in an unpaired design) could result in (design-preventable) false negative findings. Increasing the sample size will not eliminate this bias, but using a higher proportion of subjects with paired samples will, particularly if all subjects have paired samples.

There is an extreme possible study design scenario where normal tissue sample expression is obtained from a database, such as The Cancer Genome Atlas (TCGA) [14] or Genomic Data Commons (GDC) [21], to be compared to expression in study-derived tumor tissue samples. Such an approach would actually be worse than simply using unpaired data, due to the risk of substantial biasing batch effects [15], which could include platform, laboratory, and tissue ascertainment differences. While some normalization approaches may be able to successfully remove these batch effects, the corresponding data would still be comprised of entirely unpaired samples, with an accompanying loss of statistical power compared to a study design with a higher proportion of paired samples.

**Fig. 5:**
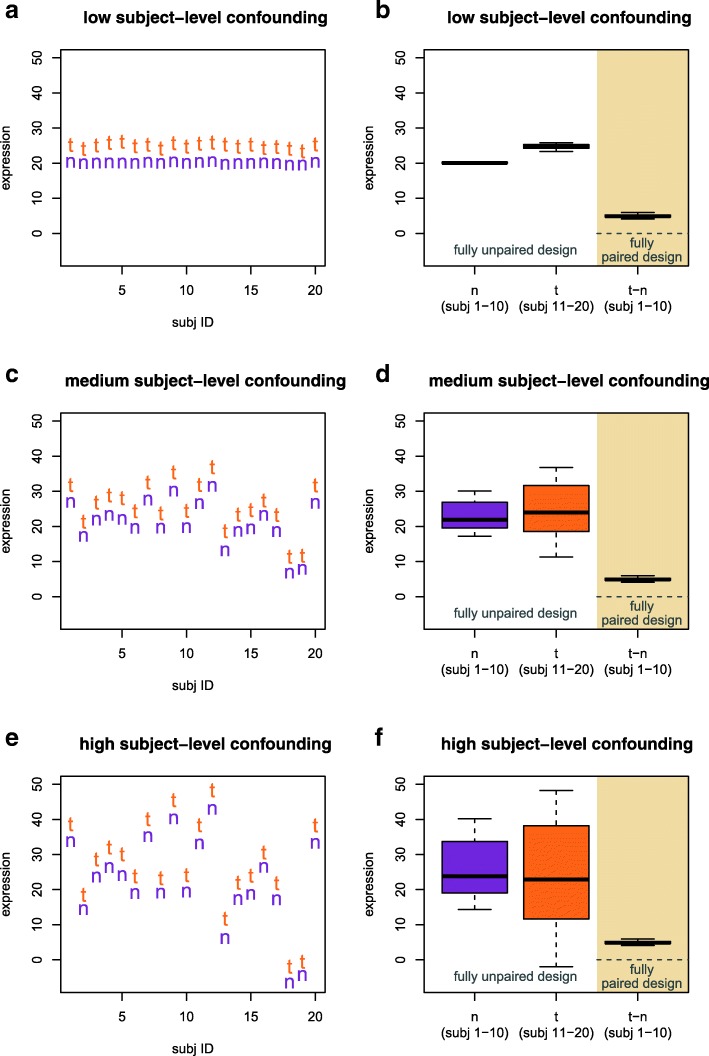
Visual example using hypothetical data to demonstrate the effect of subject-level confounding on the relative power (ability to identify a difference between t = tumor and n = normal expression) of a fully unpaired design with *N* = 20 subjects and a fully paired design with N/2 = 10 subjects

Figures 1 and 2 demonstrate that, particularly for higher levels of subject-level confounding, a higher proportion of subjects with paired samples results in higher statistical power, even so much that in many circumstances, a fully paired design with N/2 subjects can achieve higher statistical power than a totally unpaired design with N subjects. To understand how this can happen, a simple visual example with continuous data (Eq. 2 in Methods) using *N* = 20 should suffice. Figure 5a shows hypothetical expression data (both t = tumor and n = normal) for a given biomarker in each of 20 subjects. The tumor effect (i.e., the difference t-n in Fig. 5a, or TRT1-TRT2 in terms of Eq. 2) is clear, and there is very little subject-level confounding (i.e., both the tumor expression levels and the normal expression levels are relatively consistent across subjects) in Fig. 5a. Based on the same hypothetical data as in Fig. 5a, Fig. 5b shows the distribution of expression data that would be seen in a hypothetical design using normal expression (n) from subjects 1–10 and tumor expression (t) from subjects 11–20 (i.e., a fully unpaired design with N = 20 subjects); it also shows the distribution of the tumor-normal differences (t-n) that would be used in a hypothetical design using tumor and normal expression from subjects 1–10 (i.e., a fully paired design with N/2 = 10 subjects). Figure 5c and d (as well as Fig. 5e and f) are similar to Fig. 5a and b, but with increasing levels of subject-level confounding.

When subject-level confounding (${\sigma}_{Subj}^2$ in the notation explanation following Eq. 2) is low (as in Fig. 5a), the tumor and normal expression distributions (Fig. 5b) are clearly separated enough that a test of significance would be likely to detect differential expression. As subject-level confounding increases (Fig. 5c, where the tumor effect is clear but the tumor and normal expression levels are less consistent across subjects than in Fig. 5a), the shift between the tumor and normal distributions in the unpaired design (Fig. 5d) becomes less clear (and so a statistical conclusion of differential expression is less certain). For even higher subject-level confounding (Fig. 5e), the unpaired design could very well fail to identify differential expression (Fig. 5f). On the other hand, in this visual example, for every level of subject-level confounding, a fully paired design with N/2 = 10 subjects would reliably detect differential expression, since the t-n distributions in Fig. 5b, d, and f are consistently different than the dashed reference line at 0.

The key message (as visualized in Fig. 5) is that in a fully paired design, the subject-level confounding can be estimated out, leaving a clearer picture of the true underlying differential expression. Returning to Fig. 1 as an example, if the sample size is large enough and the amount of subject-level confounding (the vertical axis) is low enough, there is relatively little to gain (power-wise) by having higher proportions of subjects with paired samples (a similar conclusion to Fig. 5b). However, as the amount of subject-level confounding increases, the power impact of having a higher proportion of subjects with paired samples becomes clearer (as seen in Fig. 5d and f).

## Conclusions

There are certainly many biological systems and research questions for which paired samples are not feasible or not relevant. However, for the many biological systems and research questions where paired samples are feasible and relevant, there is clear evidence that substantial statistical power gains can be achieved at the study design stage when genomics researchers plan on collecting and using paired samples from the largest possible percentage of subjects. Failing to do so will compromise results (in terms of statistical power as well as accuracy of fold change), making it less likely that those genomics researchers will be able to detect truly differentially expressed features with meaningful fold changes. Any cost savings in a study design with unpaired samples are likely accompanied by biased and underpowered results. The greatest statistical power is achieved by having paired samples on *all* subjects; i.e. whenever paired samples are feasible and relevant, if the researcher can afford to run N samples, they should obtain paired samples on N/2 subjects.

## Methods

### Colorectal cancer study design

Study participants include all incident colon and rectal cancers between 30 and 79 years of age, who were living along the Wasatch Front in Utah or were members of the Kaiser Permanente Medical Care Program (KPMCP) in Northern California, and with primary adenocarcinoma diagnosed between October 1991 and September 1994 for colon, and between June 1997 and May 2001 for rectal. The study was approved by the Institutional Review Board of the University of Utah and at KPMCP, and all participants provided written informed consent. Numerous demographic, dietary, lifestyle, and tumor characteristic covariates were recorded for study participants, as reported previously [22, 23].

miRNA from both carcinoma and adjacent normal samples for study participants were processed (and total gene signal normalized) as described previously [22, 24], with expression quantified using the Agilent Human miRNA Microarray V19.0. miRNA expression data were thus obtained for 2006 miRNAs of which 1394 miRNAs were expressed in colorectal tissue for 1893 subjects each with paired carcinoma and adjacent normal samples.

A subset of colon cancer study participants were chosen for gene expression measurement using RNA-Seq, as described previously [25]. RNA-Seq expression data were thus obtained for 17,462 protein-coding genes, for 378 samples. These samples represent 169 subjects with paired samples, and due to QC checks, 18 normal-only subjects and 22 tumor-only subjects.

### Count expression data (RNA-Seq)

#### Model for count data

A log-linear negative binomial model is often used for biomarker count data, such as from RNA-Seq [5, 18, 26]. For a given biomarker in subject j under treatment i, the model for the count (Y_ij_) of mRNA fragments mapping to the biomarker can be parameterized as follows:

$$ {\mathrm{Y}}_{\mathrm{ij}}\sim \mathrm{NegativeBinomial}\left({\mathrm{N}}_{\mathrm{ij}},{\mathrm{p}}_{\mathrm{ij}}\right) $$

$$ \log \left(\mathrm{E}\left[{\mathrm{Y}}_{\mathrm{ij}}\right]\right)\kern0.75em =\log \left(\ {\mathrm{N}}_{\mathrm{ij}}\left(1\hbox{-} {\mathrm{p}}_{\mathrm{ij}}\right)/{\mathrm{p}}_{\mathrm{ij}}\right) $$

$$ =\log \left({\mathrm{N}}_{\mathrm{ij}}\right)+\log \left(\left(1\hbox{-} {\mathrm{p}}_{\mathrm{ij}}\right)/{\mathrm{p}}_{\mathrm{ij}}\right) $$

$$ =\log \left({\mathrm{N}}_{\mathrm{i}\mathrm{j}}\right)+\upmu +{\mathrm{TRT}}_{\mathrm{i}}+{\mathrm{CON}}_{\mathrm{j}}+{\mathrm{Subj}}_{\mathrm{j}} $$

Here, N_ij_ is the total number of counts (across all biomarkers) from subject j under treatment i, and is used as an offset (or sort of normalizing constant) to account for different total amounts of genomic material collected from different samples. The parameter p_ij_ represents the probability that any given mRNA fragment in the sample from subject j under treatment i would map to the biomarker. μ is an intercept term, TRT_i_ is the effect due to treatment level i, and Subj_j_ is the effect due to subject j.

Each subject j has a possibly confounding factor (or combination of factors, CON) that will affect the value of both their normal and their tumor expression values. (This confounding factor effect is *critical*, and could result from a combination of dietary, lifestyle, genetic, and other unknown factors, all with biomarker-specific confounding effects.) This factor is confounded with the Subject effect in the model, so the model can be rewritten as

1 $$ \log \left(\mathrm{E}\left[{\mathrm{Y}}_{\mathrm{i}\mathrm{j}}\right]\right)=\log \left({\mathrm{N}}_{\mathrm{i}\mathrm{j}}\right)+\upmu +{\mathrm{TRT}}_{\mathrm{i}}+{\mathrm{Subj}}_{\mathrm{j}} $$

TRT is a fixed effect (with only two levels of interest, say tumor and normal), and Subj is a random effect (with many subjects of interest, including those not in the study). The presence of both fixed and random effects makes this a “mixed” model, and the non-normal distribution of F with a linear model for the expected value of Y (after log link function transformation) makes this a “generalized linear” mixed model.

Because Subj is a random effect, the values of Subj_j_ are assumed to be independent and identically distributed Normal(0, ${\sigma}_{Subj}^2$). If there is a greater confounding effect (such as perhaps some subjects’ diet or lifestyle factors affect their expression values), this will result in larger Subj_j_ values (positive or negative) for those subjects in Eq. 1 above. These larger values (i.e., larger subject effects) will be reflected by a larger variance among the subject effects, and quantified by a larger value of ${\sigma}_{Subj}^2$ . In this way, the strength of the confounding factor is quantified by the between-subject variance component ${\sigma}_{Subj}^2$. This variance component, along with treatment effect size and a model “scale” parameter, need to be estimated to obtain useful power approximations. (Regarding the scale parameter, the glmer.nb function in R produces what it calls $\widehat{\theta}$, and PROC GLIMMIX in SAS uses $\sqrt{\widehat{k}}=1/\sqrt{\widehat{\theta}}$; see lme4 [27] and GLIMMIX [28] documentation for details.)

#### Estimates from previous count data

A negative binomial model for biomarker count data can be fit by popular R packages such as DESeq2 [18] and edgeR [26]; while these packages feature pooling of information across biomarkers and have been shown to be powerful, they do not allow for random effects. Instead, model (1) above was fit using the glmer.nb function of the lme4 package [27] for R [20], for each of 17,462 protein-coding genes in our CRC data, because this approach is specifically designed for both fixed and random effects. (This took 54 h computation time, optimized to about 3 h real time using the batch computing resources of the Center for High Performance Computing at the University of Utah.)

In these data, many thousands of genes were significantly differentially expressed (even after multiplicity corrections), but those are not of greatest interest in this manuscript. Instead, it is most relevant to note that even for very small TRT effects, statistical significance was noted. Based on the results for our CRC data, a range of ${\sigma}_{Subj}^2$ values from 0.5 to 2.5 (on the 10th root scale) was considered most reasonable. (The 10th root transformation of ${\sigma}_{Subj}^2$ was chosen for visualization purposes as the strength of the subject-level confounding factor in Fig. 1; less than 2 % of genes had subject variance component estimates of zero.) While there was also a range of scale estimates, the effect of the scale parameter on statistical power is actually closely tied to that of the treatment effect TRT (which is the log of the fold change; holding other variance parameters constant, the scale parameter and the fold change go hand-in-hand: for a larger scale parameter, it takes a larger fold change to achieve the same statistical power). For this reason, we set the scale parameter value at 1.15 in our power approximation (which was the approximate average for the scale parameter for significantly differentially expressed genes), and focus on statistical power results for detecting a subtle fold change of 1.25 (equivalent to a TRT effect, or log fold-change, of 0.22).

#### Power approximation for count data

The probability distribution method [29] is a very flexible tool for approximate power calculations in mixed models. It is implemented using PROC GLIMMIX in SAS, and takes as arguments the variance components for model (1) above as well as an “exemplary” data set exhibiting a given TRT effect for a given study design (including total number of samples N and percent of subjects with paired samples). The SAS code with the implementation of this method for this manuscript is included as Additional file 1.

The probability distribution method was chosen after attempts at the simulation method for power approximation [29] for count data proved to be computationally unfeasible. Briefly, the generalized linear mixed model is more computationally expensive than a simple general mixed model, so much so that simulating sufficient data to approximate power on the scale needed for this manuscript would have taken several months’ worth of real computation time, even after utilizing batch computing resources. By comparison, while the actual SAS code in Additional file 1 took some time to develop and debug, the probability distribution method requires only 5–10 min’ worth of computation time to produce the contour plots in Fig. 1 (depending on the number of samples N).

### Continuous expression data (microarray)

#### Model for continuous data

A linear mixed model can be used for continuous expression data, such as for cDNA [30], mRNA [31–33], or miRNA [34] microarrays, as well as for RT-PCR data [35]. For a given biomarker in subject j under treatment i, the log-scale expression level of a given biomarker can be parameterized as follows:

$$ {\mathrm{Y}}_{\mathrm{i}\mathrm{j}}=\upmu +{\mathrm{TRT}}_{\mathrm{i}}+{\mathrm{CON}}_{\mathrm{j}}+{\mathrm{Subj}}_{\mathrm{j}}+{\upvarepsilon}_{\mathrm{i}\mathrm{j}} $$

Here, *i* = 1,2 (for two ‘treatment’ conditions such as tumor and normal), and j = 1, …, #subjects. This model assumes that the data have been appropriately preprocessed and normalized. As in the model for count data above, each subject j has a possibly confounding factor (or combination of factors, CON) that will affect the expression of both their normal and their tumor expression values. This factor is confounded with the Subject effect in the model, so the model can be rewritten as

2 $$ {\mathrm{Y}}_{\mathrm{i}\mathrm{j}}=\upmu +{\mathrm{TRT}}_{\mathrm{i}}+{\mathrm{Subj}}_{\mathrm{j}}+{\upvarepsilon}_{\mathrm{i}\mathrm{j}} $$

If the biomarker is not differentially expressed, then TRT_1_ = TRT_2_ = 0. The random subject (or confounding factor) effects are assumed to follow a Normal(0, ${\sigma}_{Subj}^2$) distribution (with ${\sigma}_{Subj}^2$ representing the strength of the confounding factor effect), and the random error terms are assumed to follow a Normal(0,*σ*^2^) distribution.

#### Estimates from previous continuous data

Using the lmer function of the R package lme4 [27], model (2) was fit to each of 1394 miRNAs in our CRC data. (This took less than 3 min on a desktop computer.) From this model fit, variance components ${\sigma}_{Subj}^2$ and *σ*^2^ were both estimated, along with the treatment effect. The estimated ${\sigma}_{Subj}^2$ essentially ranged from 0 to 1.5 (the strength of the subject-level confounding factor in Fig. 2 is ${\sigma}_{Subj}^2$), and *σ*^2^ was roughly linearly associated with ${\sigma}_{Subj}^2$, with a slope of 1.73. There was no real association between either of the variance components and the TRT effect, which ranged to ±2, but with a majority ranging to ±0.5. A fold change of 1.5 was chosen as a desired target for power approximation, corresponding to a TRT effect (or log fold-change) of approximately 0.41.

#### Power approximation for continuous data

The probability distribution method [29] was used for approximate power calculations across a range of ${\sigma}_{Subj}^2$ values, and for various percentages of subjects with paired samples, using the parameter estimate ranges described in the previous paragraph. The SAS code with the implementation of this method for continuous data in this manuscript is included as Additional File 2.

In addition, the simulation method [29] was implemented to verify the general trends in the power contours (shown for the probability method in Fig. 2) as well as false discovery rate (FDR) control [19]. Briefly, this method involves simulating many data sets exhibiting the variance components and treatment effects of interest, and then fitting the appropriate model to those data sets. The power is approximated by averaging across the many data sets’ results. Using different combinations of N (total number of samples to be run; 50, 100, 200, and 500), ${\sigma}_{Subj}^2$ (strength of confounding factor; ranging from 0 to 2), and percentage of subjects with paired samples (ranging from 0 to 100), we simulated data according to model (2). At each combination setting, 1000 biomarkers were simulated 100 times; each time there were 20 biomarkers selected to be differentially expressed, with non-zero TRT effects randomly drawn between 1 and 2. Within each set of 1000 biomarkers simulated, the FDR was controlled at .05 in the test of differential expression (testing the TRT effect in the model). Across the 100 simulations at each parameter combination, the estimated power and FDR were averaged. These simulations took about 38 days computation time, optimized to about 6 h real time using the batch computing resources of the Center for High Performance Computing at the University of Utah.

### Paired vs unpaired design: RNA-Seq power comparison in colon cancer study

For demonstration purposes of the practical effect of full vs. partial (or no) pairing, we randomly selected 100 subjects (from 169) with paired samples in the RNA-Seq data, and fit model (1) on each of the 17,462 protein-coding genes. The test for differential expression (of the TRT effect) in this fully paired design (with 200 total samples) yielded a *p*-value for each feature, and these were adjusted to control the false discovery rate (FDR) [19].

Unpaired design 1 consisted of using the same data as in this paired design, for the same 100 subjects. A variation of model (1) was fit, excluding the subject random effect, which is equivalent to ignoring the pairing. Again FDR-adjusted *p*-values were obtained for each of the 17,462 protein-coding genes.

Unpaired design 2 used data for the same 100 subjects, but with 50 of those subjects randomly selected to be tumor-only and the other 50 to be normal-only (i.e., only their tumor or only their normal samples were used). Then of the subjects not among those 100, 50 others were randomly selected (from the 22 normal-only and the 69 unselected paired) to be tumor-only and another 50 (from the original 18 normal-only and those still not selected of the 69 paired) to be normal-only. Data for these 200 total samples were used to fit a variation of model (1), excluding the subject random effect (due to a total lack of pairing in this design), and again FDR-adjusted p-values were obtained for each of 17,462 protein-coding genes.

One way to look at these three designs is as follows. The paired design is intended to represent the ideal, with every subject providing paired samples and the analysis accounting for the pairing. Unpaired design 1 is intended to represent partially lost potential, with every subject providing paired samples but the analysis not accounting for the pairing. Unpaired design 2 is intended to represent fully lost potential, with no paired samples for any subject.

To demonstrate the potential impact of accounting for subject-level covariates in an unpaired design, we again ran a variation of model (1) on both unpaired design 1 and unpaired design 2, but instead of the subject random effect we included a few covariates as fixed effects (or continuous covariate in the case of age at diagnosis). The covariates included tumor site (proximal or distal colon), tumor SEER summary stage (distant, local, regional, insitu, or unknown), age at diagnosis, MSI tumor status, and sex. These covariates were chosen only for demonstrative purposes as potential subject-level confounding factors that have previously been shown to be associated with expression levels in colorectal cancer [23]. Table 1 summarizes these covariates in the paired and two unpaired designs.

**Table 1:** Summary of covariates in paired and unpaired designs for RNA-Seq colon data

	Paired Design and Unpaired Design 1	Unpaired Design 2
Tumor Site	Proximal: 100 Distal: 100	Proximal: 100 Distal: 100
SEER stage	Distant: 24 Local: 72 Regional: 102 Unknown: 2	Distant: 25 Local: 70 Regional: 103 Unknown: 1 Insitu: 1
Age at diagnosis (years)	Mean 63.9, SD 10.0	Mean 64.9, SD 10.1
MSI tumor status	MSS: 164 MSI: 36	MSS: 166 MSI: 34
Sex	Female: 106 Male: 94	Female: 107 Male: 93

## Additional files

Additional file 1: SAS code to implement the probability distribution method for calculating approximate power with count expression (such as RNA-Seq) data (SAS 9 kb)

Additional file 2: SAS code to implement the probability distribution method for calculating approximate power with continuous expression (such as miRNA) data (SAS 9 kb)

Additional file 3: Approximate power contours for continuous data, such as miRNA, based on the (computationally expensive) simulation method. Contours are given for the average and median power across 100 simulations. In an effort to quantify the amount of variability across the 100 simulations, contours are also given for the standard deviation (SD) and range (max minus min) of power across the 100 simulations. (PDF 68 kb)

Additional file 4: Approximate false discovery rate contours for continuous data, such as miRNA, based on the (computationally expensive) simulation method. Contours are given for the average and median FDR across 100 simulations. In an effort to quantify the amount of variability across the 100 simulations, contours are also given for the standard deviation (SD) and range (max minus min) of the FDR across the 100 simulations. (PDF 101 kb)
