# Supplementary material for: Power in pairs: assessing the statistical value of paired samples in tests for differential expression
Source: BMC Genomics. 2018 Dec 20;19:953. doi: 10.1186/s12864-018-5236-2 (PMC6302489; doi:10.1186/s12864-018-5236-2)
Supplement: Supplementary file 3 — Approximate power contours for continuous data, such as miRNA, based on the (computationally expensive) simulation method. Contours are given for the average and median power across 100 simulations. In an effort to quantify the amount of variability across the 100 simulations, contours are also given for the standard deviation (SD) and range (max minus min) of power across the 100 simulations. (PDF 68 kb) [file 12864_2018_5236_MOESM3_ESM.pdf]

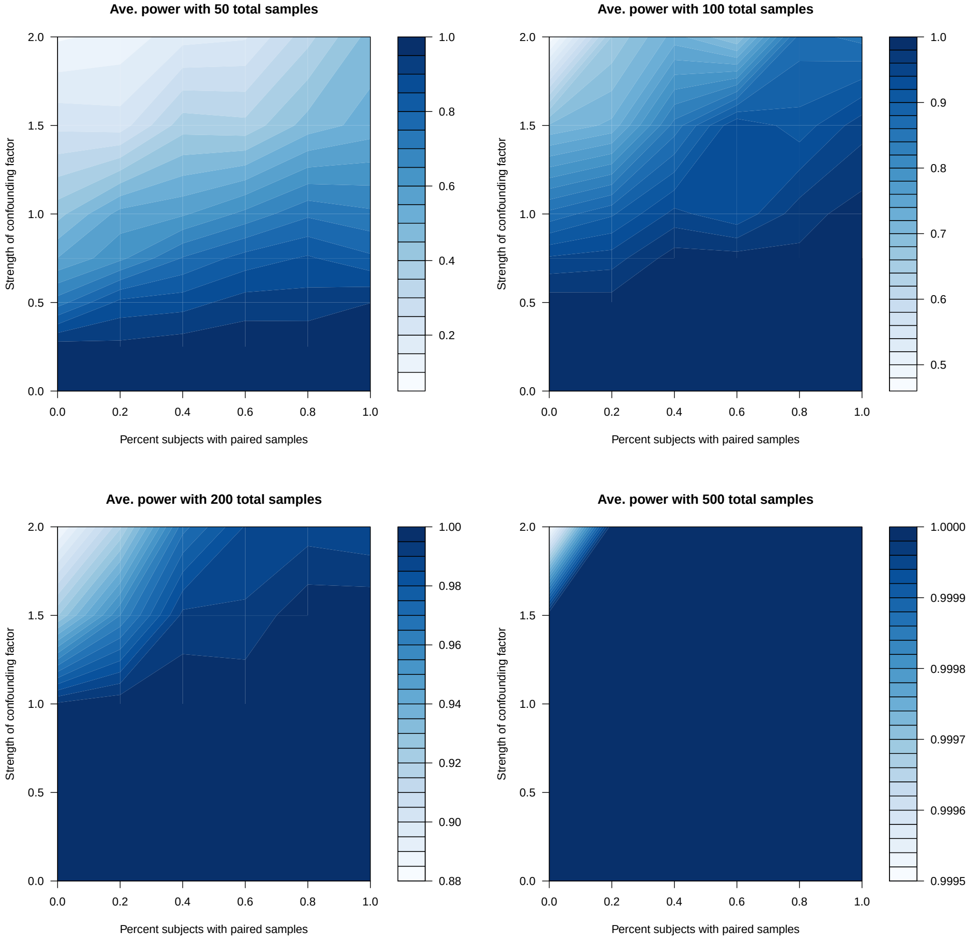

Ave. power with 50 total samples
Ave. power with 100 total samples
Ave. power with 200 total samples
Ave. power with 500 total samples
Strength of confounding factor
Percent subjects with paired samples
0.0
0.5
1.0
1.5
2.0
0.2
0.4
0.6
0.8
0.9
0.7
0.5
1.00
0.98
0.96
0.94
0.92
0.90
0.88
1.0000
0.9999
0.9998
0.9997
0.9996
0.9995

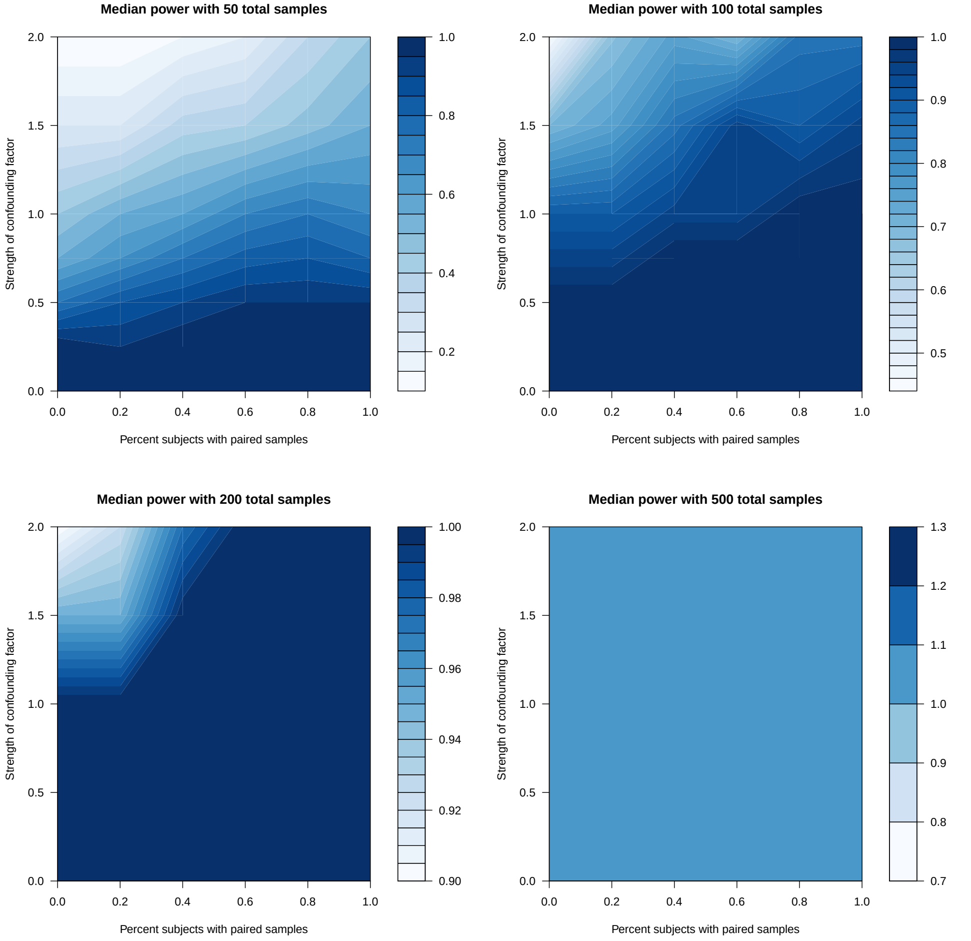
Median power with 50 total samples
Strength of confounding factor
Percent subjects with paired samples
0.0 0.5 1.0 1.5 2.0
0.0 0.2 0.4 0.6 0.8 1.0
0.2 0.4 0.6 0.8 1.0
Median power with 100 total samples
Strength of confounding factor
Percent subjects with paired samples
0.0 0.5 1.0 1.5 2.0
0.0 0.2 0.4 0.6 0.8 1.0
0.5 0.6 0.7 0.8 0.9 1.0
Median power with 200 total samples
Strength of confounding factor
Percent subjects with paired samples
0.0 0.5 1.0 1.5 2.0
0.0 0.2 0.4 0.6 0.8 1.0
0.90 0.92 0.94 0.96 0.98 1.00
Median power with 500 total samples
Strength of confounding factor
Percent subjects with paired samples
0.0 0.5 1.0 1.5 2.0
0.0 0.2 0.4 0.6 0.8 1.0
0.7 0.8 0.9 1.0 1.1 1.2 1.3

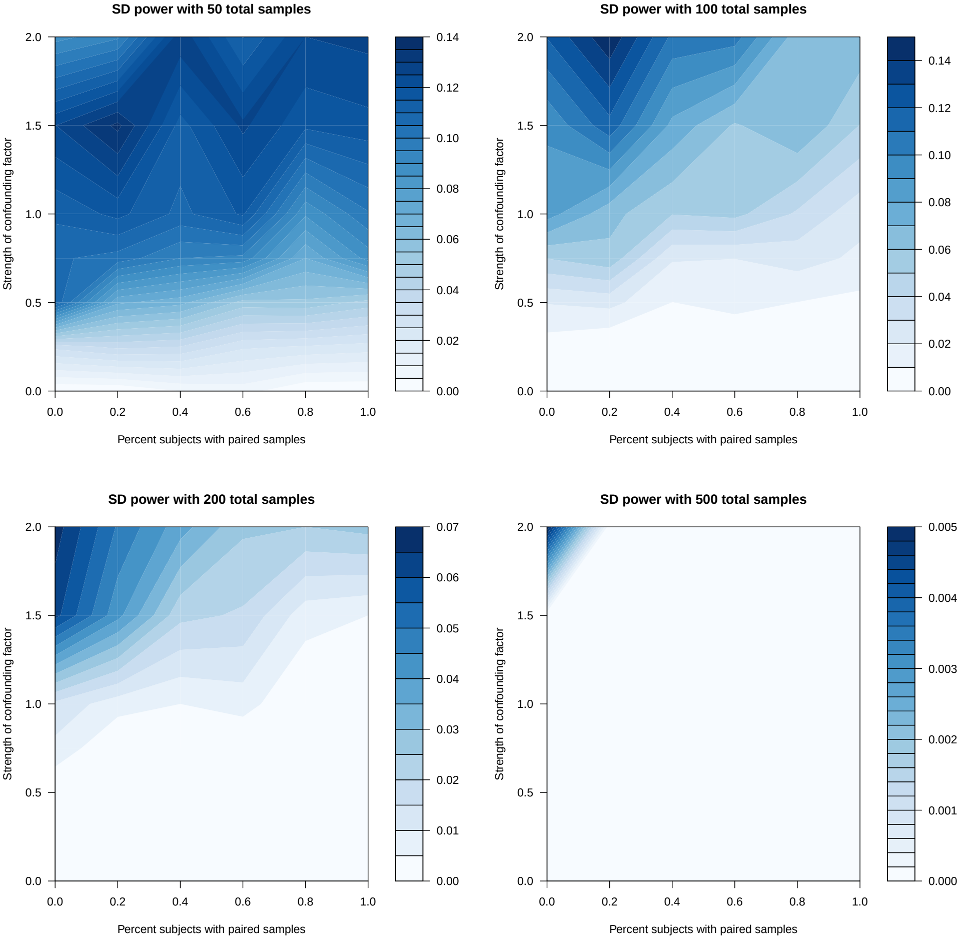
SD power with 50 total samples
SD power with 100 total samples
SD power with 200 total samples
SD power with 500 total samples
Strength of confounding factor
Percent subjects with paired samples
0.0
0.2
0.4
0.6
0.8
1.0
0.5
1.5
2.0
0.00
0.02
0.04
0.06
0.08
0.10
0.12
0.14
0.01
0.03
0.05
0.07
0.000
0.001
0.002
0.003
0.004
0.005

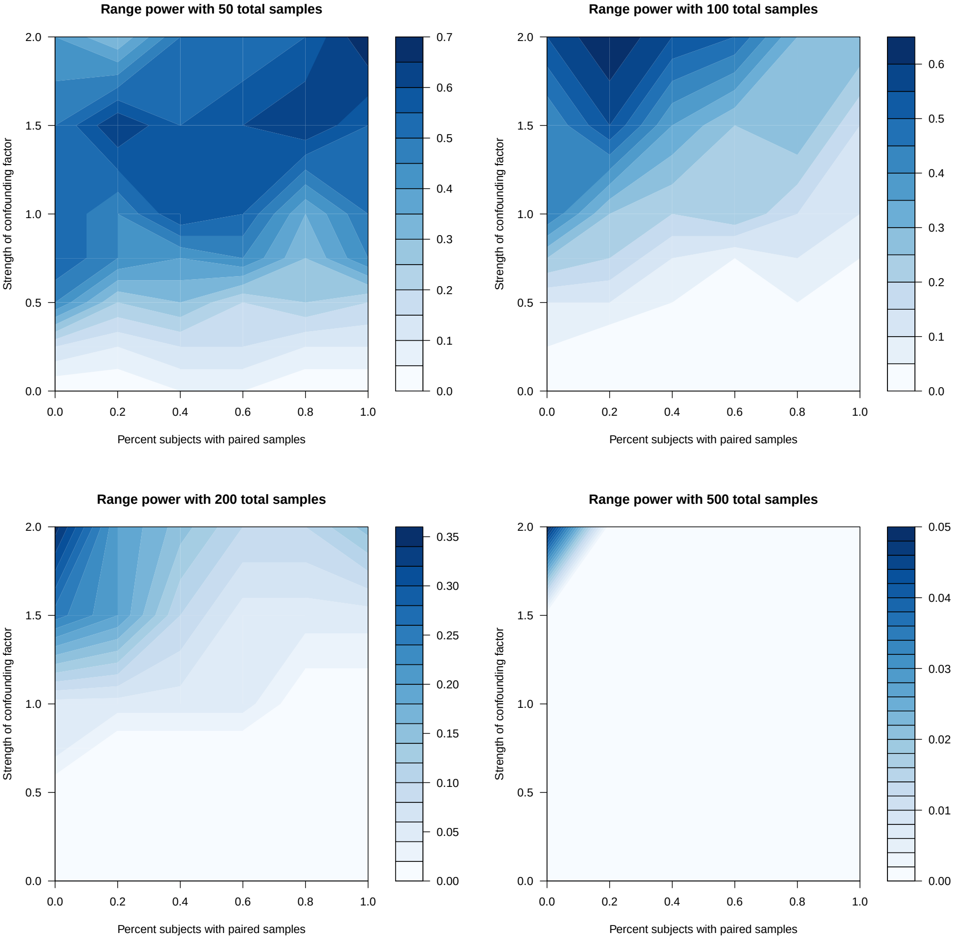
Range power with 50 total samples
Range power with 100 total samples
Range power with 200 total samples
Range power with 500 total samples
Strength of confounding factor
Percent subjects with paired samples
0.0
0.5
1.0
1.5
2.0
0.0
0.2
0.4
0.6
0.8
1.0
0.0
0.1
0.2
0.3
0.4
0.5
0.6
0.7
0.00
0.05
0.10
0.15
0.20
0.25
0.30
0.35
0.00
0.01
0.02
0.03
0.04
0.05
