# Supplementary material for: Power in pairs: assessing the statistical value of paired samples in tests for differential expression
Source: BMC Genomics. 2018 Dec 20;19:953. doi: 10.1186/s12864-018-5236-2 (PMC6302489; doi:10.1186/s12864-018-5236-2)
Supplement: Supplementary file 4 — Approximate false discovery rate contours for continuous data, such as miRNA, based on the (computationally expensive) simulation method. Contours are given for the average and median FDR across 100 simulations. In an effort to quantify the amount of variability across the 100 simulations, contours are also given for the standard deviation (SD) and range (max minus min) of the FDR across the 100 simulations. (PDF 101 kb) [file 12864_2018_5236_MOESM4_ESM.pdf]

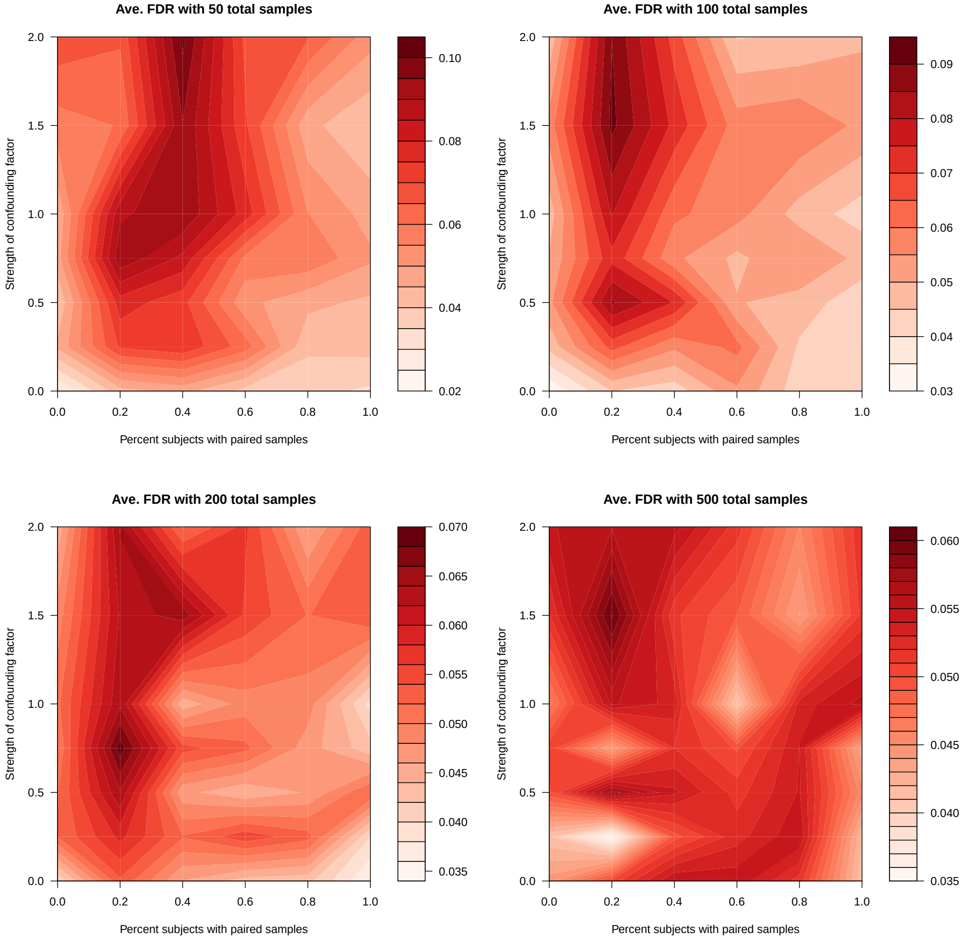

Ave. FDR with 50 total samples
Ave. FDR with 100 total samples
Ave. FDR with 200 total samples
Ave. FDR with 500 total samples
Strength of confounding factor
Percent subjects with paired samples
0.0
0.5
1.0
1.5
2.0
0.2
0.4
0.6
0.8
0.02
0.04
0.06
0.08
0.10
0.03
0.05
0.07
0.09
0.035
0.040
0.045
0.050
0.055
0.060
0.065
0.070

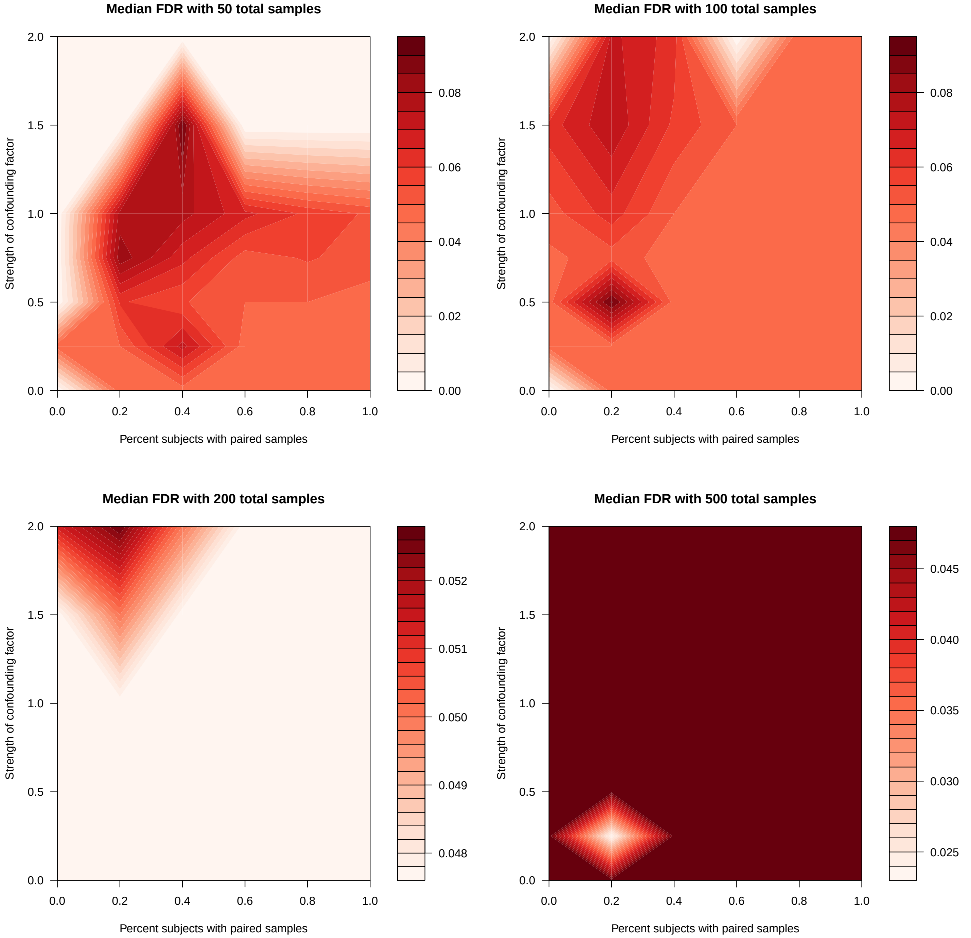

Median FDR with 50 total samples
Median FDR with 100 total samples
Median FDR with 200 total samples
Median FDR with 500 total samples
Strength of confounding factor
Percent subjects with paired samples
0.0
0.5
1.0
1.5
2.0
0.2
0.4
0.6
0.8
0.00
0.02
0.04
0.06
0.08
0.048
0.049
0.050
0.051
0.052
0.025
0.030
0.035
0.040
0.045

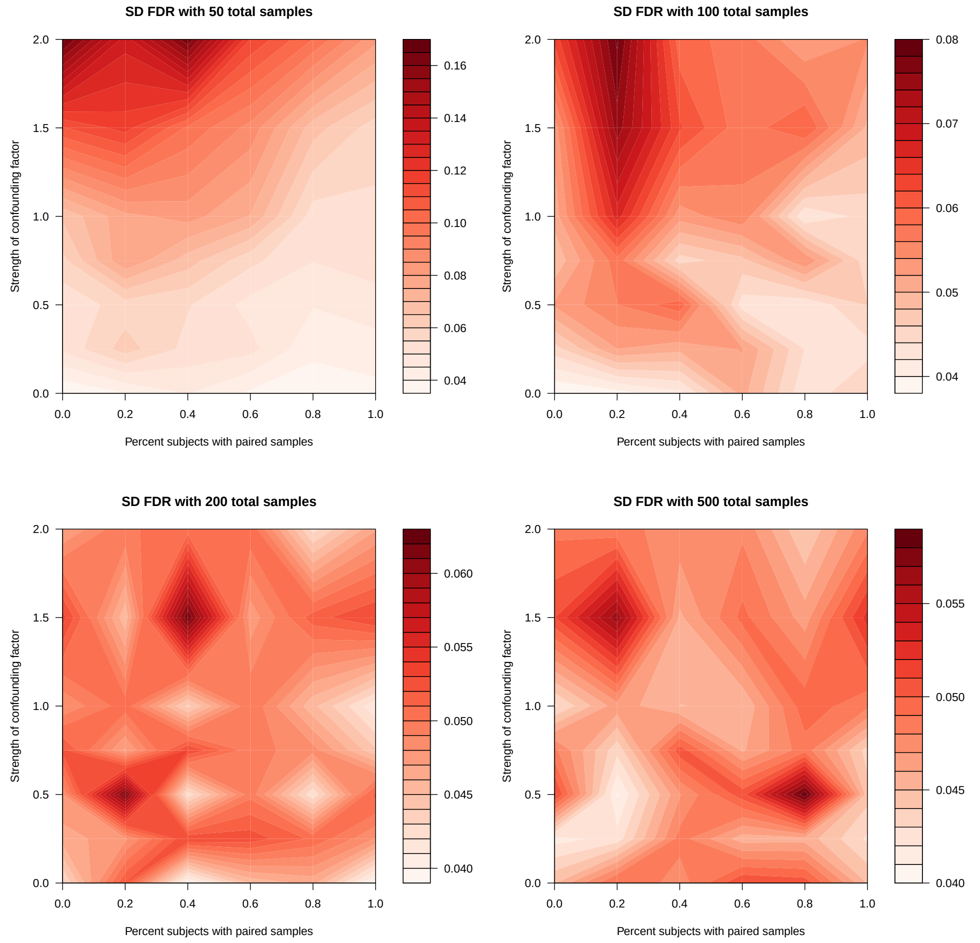
SD FDR with 50 total samples
SD FDR with 100 total samples
SD FDR with 200 total samples
SD FDR with 500 total samples
Strength of confounding factor
Percent subjects with paired samples
0.0
0.5
1.0
1.5
2.0
0.2
0.4
0.6
0.8
0.16
0.14
0.12
0.10
0.08
0.06
0.04
0.07
0.05
0.060
0.055
0.050
0.045
0.040

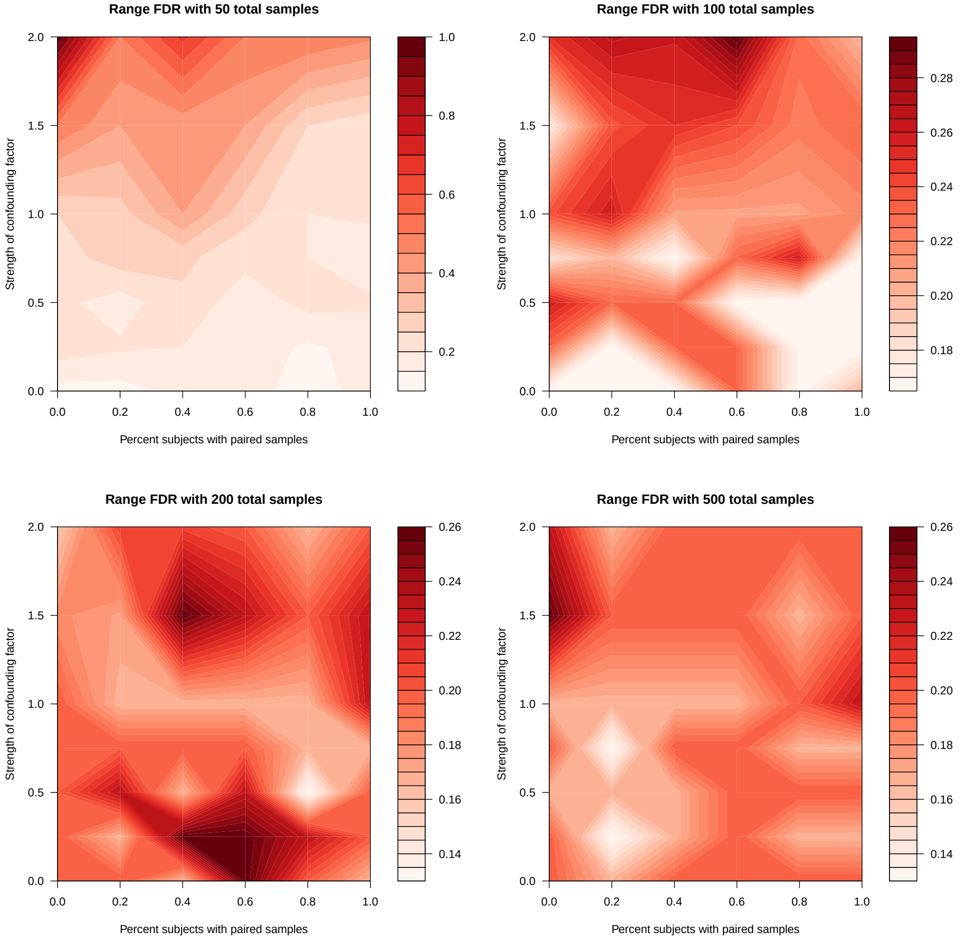

Range FDR with 50 total samples
Range FDR with 100 total samples
Range FDR with 200 total samples
Range FDR with 500 total samples
Strength of confounding factor
Percent subjects with paired samples
0.0
0.2
0.4
0.6
0.8
1.0
0.5
1.5
2.0
0.18
0.20
0.22
0.24
0.26
0.28
0.14
0.16
